# Nutcracker Syndrome in Children: Role of Doppler Ultrasonographic Indices in Detecting the Pattern of Symptoms

**DOI:** 10.3390/jcm7080214

**Published:** 2018-08-13

**Authors:** Hulya Nalcacioglu, Meltem Ceyhan Bilgici, Demet Tekcan, Gurkan Genc, Yakup Bostanci, Yarkin Kamil Yakupoglu, Saban Sarikaya, Ozan Ozkaya

**Affiliations:** 1 Pediatric Nephrology Department, Ondokuz Mayis University Faculty of Medicine, 55220 Samsun, Turkey; demettekcan@yahoo.com (D.T.); gencdoc@yahoo.com (G.G.); 2 Pediatric Radiology Department, Ondokuz Mayis University Faculty of Medicine, 55220 Samsun, Turkey; mceyhan@omu.edu.tr; 3 Urology Department, Ondokuz Mayis University Faculty of Medicine, 55220 Samsun, Turkey; yakup.bostanci@omu.edu.tr (Y.B.); ykamil@omu.edu.tr (Y.K.Y.); 4 Pediatric Urology Department, Ondokuz Mayis University Faculty of Medicine, 55220 Samsun, Turkey; sarikaya@omu.edu.tr; 5 Pediatric Nephrology Department, Istinye University Faculty of Medicine, 34010 Istanbul, Turkey; ozanozkaya@yahoo.com

**Keywords:** left renal vein, nutcracker syndrome, doppler ultrasonography, pediatrics

## Abstract

The purpose of this study was to evaluate the clinical characteristics of 44 pediatric patients who were diagnosed as having nutcracker syndrome (NCS). We also investigated the left renal vein Doppler ultrasonography (DUS) results, to determine whether or not there was an association between clinical symptoms and DUS findings among these patients. The clinical data from 44 pediatric patients who were diagnosed as having NCS from January 2008 to December 2015 were retrospectively reviewed. We grouped the patients according to the presenting symptoms as symptomatic (loin pain; macroscopic hematuria or both) and non-symptomatic (microscopic hematuria and proteinuria were detected incidentally) and evaluated the left renal vein DUS indices in these two groups separately. Asymptomatic NCS was found in 27 (61.4%) patients; 21 (47.7%) of whom were admitted for the evaluation of proteinuria. The most frequent presenting symptoms were left flank pain (20.5%) and macroscopic hematuria (13.6%); and 2 (4.5%) patients presented with a combination of left flank pain and macroscopic hematuria. The mean ratio of the diameter of the hilar portion of the left renal vein (LRV) to that of the aortomesenteric portion was 4.36 ± 1.55. The mean ratio of the peak velocity (PV) between the two sites of the LRV was 7.32 ± 2.68 (3.1–15.6). The differences in the ratio of the diameters were statistically significant between the two groups and significantly higher in children with asymptomatic NCS (*p* = 0.025). The PV ratios of the LRV (*p* = 0.035) were significantly higher in asymptomatic children with NCS than in the symptomatic group. Our study identifies that increased compression ratio of the LRV entrapment is most observed in orthostatic proteinuria and microscopic hematuria.

## 1. Introduction

Nutcracker syndrome (NCS) results in left renal venous hypertension due to compression of the left renal vein (LRV) between the superior mesenteric artery (SMA) and aorta (anterior NCS), or between the aorta and vertebral column (posterior NCS) [1,2,3]. Nutcracker phenomenon (NCP) and NCS are usually used synonymously in the literature [4,5]; however, NCP is an asymptomatic finding of LRV entrapment, whereas NCS is a complex of clinical signs and symptoms that result from NCP [2,3,5,6].

NCS typically presents as pelvic pain, flank pain, hematuria, gonadal varices (varicocele or ovarian vein syndrome), orthostatic proteinuria, and orthostatic intolerance [5,6,7,8,9,10,11,12]. It is hypothesized that the compression increases venous pressure within the renal circulation, which affects the left kidney by causing congestion, and sometimes through collateral circulation formation, resulting in micro- or macrohematuria [2,3,6]. Orthostatic proteinuria is the another commonly reported sign in NCS [8,9,10,11]. The prevalence of this condition is not exactly known because of an undetermined diagnostic criteria and variability of symptoms in presentations. The diagnosis of NCS is confirmed using imaging techniques such as Doppler ultrasonography (DUS), computed tomography angiography (CTA), magnetic resonance angiography (MRA), and retrograde venography [2,3,6,13]. The sensitivity (69–90%) and specificity (89–100%) of DUS makes it a helpful noninvasive diagnostic tool, especially in the presence of color flow in collateral veins [2,8,12,14,15,16]. DUS cutoff values of childhood NCS could vary from 3.7–4.0 to 4.7–4.8 [8,14,17,18]. Cheon et al. [17] suggested that cut-off values of more than 4.7 for the ratio of peak velocity (PV) of the aortomesenteric (AM) segment to the hilar portion for the LRV could be used as diagnostic criterion for NCS in children.

To further characterize the clinical and imaging characteristics of patients with NCP, we performed a retrospective review of patients diagnosed with this condition at our institution. We then divided the sample into two clinical cohorts (symptomatic and asymptomatic) and compared their DUS results to determine whether or not there was an association between clinical symptoms and DUS findings among these patients.

## 2. Material and Methods

We retrospectively reviewed 44 cases of anterior NCS (25 girls and 19 boys; age range, 7–18 years) from January 2008 to December 2015 at our Pediatric Nephrology Department in Ondokuz Mayis University Faculty of Medicine. The clinical characteristics of the patients, main symptoms at admission, positive findings on physical examination, radiologic findings (DUS, CTA), laboratory examinations (urine analysis, 24-hour protein excretion), and clinical course were analyzed retrospectively.

The diagnosis of NCS based on the presence of the clinical (left flank pain, macroscopic hematuria) and laboratory findings (macro- or microscopic hematuria and proteinuria), as well as the findings of left renal DUS results. The persistent presence of greater than 5 red cells/mm^3^ in uncentrifuged urine is defined as hematuria. Proteinuria was considered as protein levels between 4 and 40 mg/m^2^/24 hour. Orthostatic proteinuria is characterized by the presence of protein in urine samples collected in the upright position and its absence in samples collected in the supine position. Urine analysis, urine phase contrast microscopy, urine culture, blood pressure, renal function, blood examinations including a complete blood count, electrolytes, complement levels, and urinary Ultrasonography (USG) were normal in all patients, and we excluded any underlying diseases.

Renal DUS examinations were performed for all patients while they were in the supine position using a Toshiba Aplio XG SSA-790A (Toshiba Medical Systems Corporation, Otowara, Japan) USG device with a convex probe (frequency 3.5 MHz). DUS data were obtained by a pediatric radiologist who was blinded as to the probable NCS diagnoses of the patients. The antero-posterior (AP) diameter and the PV of the LRV were measured at the hilar and AM portions and ratios of the diameter and PV between the 2 sites were calculated. We applied the cut-off value of ≥4.7 for the PV ratio of the LRV between the AM and hilar portions according to published studies in children with NCS [12,17].

We grouped the patients according to the presenting clinical symptoms as symptomatic (loin pain, macroscopic hematuria or both) and non-symptomatic (microscopic hematuria and proteinuria were detected incidentally), and evaluated the LRV DUS indices in these two groups separately. The ethical board of our hospital approved this retrospective study in accordance with the 1975 Helsinki Declaration (Decision no. 2011/443).

### Statistical Analysis

Analyses were performed using the Statistical Package for the Social Sciences 22.0 (SPSS IBM Corp, Armonk, NY, USA). Compatibility of variables were investigated using visual (histogram and probability diagrams) and analytical (Shapiro-Wilk) tests. The characteristics of the patients were determined using descriptive statistics. Parameters compatible with normal distribution were defined as mean ± standard deviations (SD), and parameters that did not fit normal distribution were defined as median and distribution (lower-upper limit). The comparisons of proportions were performed using the Chi-square test. For the comparisons between the asymptomatic and the symptomatic group, the independent samples *t*-test was used for parameters with normal distribution, and the Mann-Whitney U-test was used for parameters with non-normal distribution. *P* values less than 0.05 were considered as statistically significant.

## 3. Results

We retrospectively investigated 44 children who were diagnosed as having NCS from 2008 to 2015, of which 19 (43.2%) were male and 25 (56.8%) were female. The mean age of the study group was 12.8 ± 2.79 years (range, 7 to 18 years). Asymptomatic NCS was found in 27 (61.4%) patients, of whom 21 (47.7%) were admitted for the evaluation of proteinuria. Seventeen (38.6%) patients had symptomatic NCS. The most frequently presented symptoms were left flank pain (20.5%) and macroscopic hematuria (13.6%), and 2 (4.5%) patients presented with the combination of left flank pain and macroscopic hematuria. No significant differences were found between the asymptomatic and symptomatic group in terms of age and sex (*p* = 0.787, *p* = 0.096, respectively). The median body mass index (BMI) values were 16.4 kg/m^2^ (range, 14.2–26.8 kg/m^2^). The median 24 h protein excretion was 13 mg/m^2^/hour (range 1.6–42 mg/m^2^/hour). The median time until NCS diagnosis was 2.5 months (range, 1–60 months). All patients underwent LRV DUS as the initial diagnostic test with suspected nutcracker syndrome. We successfully obtained Doppler spectra from the LRV at the aortomesenteric and hilar portions in 36 of 44 patients. Computed Tomography Angiography (CTA) was used in 8 patients for the diagnostic purposes along with DUS due to visualize the AM portions of the LRV. DUS and CTA findings showed anterior NCS in all patients.

**Figure 1:**
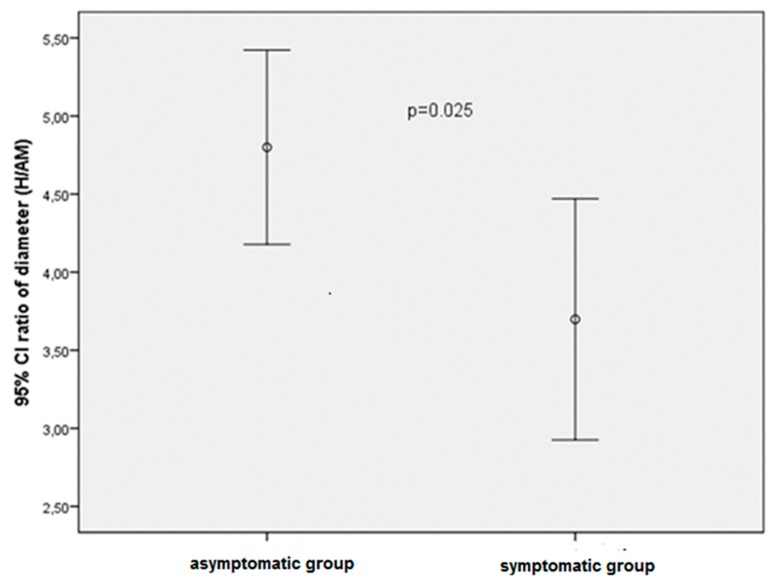
Comparison of 95% confidance intervals (CI) of diameter ratios (hilar/aortomesenteric portion) levels in asymptomatic and symptomatic patients.

The clinical, presenting features, and LRV DUS indices of the study group are shown in Table 1. In the study group, the mean diameter of the LRV at the hilar and the median diameter of AM portions were 8.43 ± 3.25 mm and 2 mm (range, 1.1–6.1 mm), respectively (ratio: 4.36 ± 1.55). The mean PV of the LRV at the AM and hilar portion was 144.9 ± 56.73 cm/s and 19.72 ± 5.09 cm/s, respectively (ratio: 7.32 ± 2.68) (Table 1). Table 2 shows the indices of the LRV DUS in the asymptomatic and symptomatic groups. The differences in the diameter at each site were not statistically significant between the two groups (*p* = 0.215 for hilar, *p* = 0.103 for the AM portion). The mean ratios of the diameter at the hilar portion to the AM portion was 4.79 ± 1.47 and 3.69 ± 1.45 in the asymptomatic and symptomatic groups, respectively. The differences in the ratio of the diameters were statistically significant between the two groups, and significantly higher in children with asymptomatic NCS (*p* = 0.025; Table 2, Figure 1).

**Figure 2:**
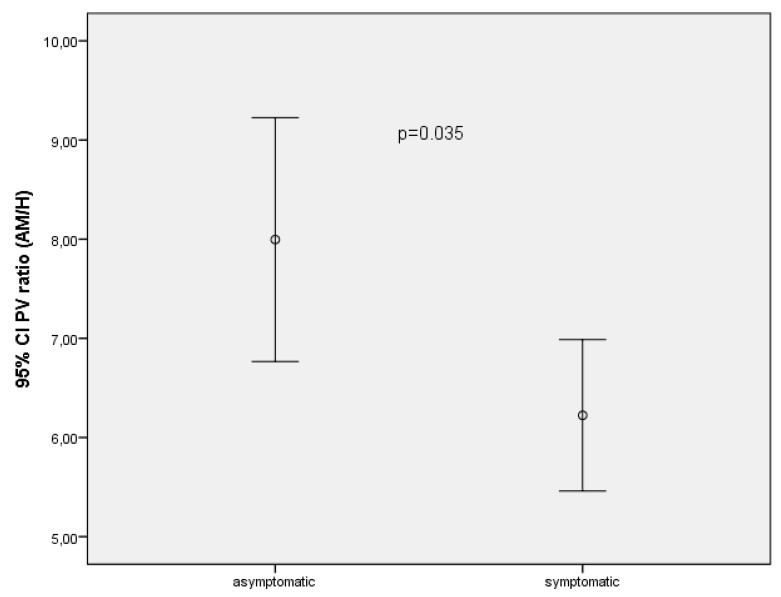
Comparison of 95% confidance intervals (CI) of peak velocity ratios (hilar/aortomesenteric portion) levels in asymptomatic and symptomatic patients.

In the asymptomatic group, the mean PV of the LRV was 150.08 ± 63.53 cm/s at the AM portion and 18.77 ± 5.22 cm/s at the hilar portion. In the symptomatic group, the mean PV of the LRV was 135.93 ± 43.01 cm/s at the AM portion and 21.36 ± 4.55 cm/s at the hilar portion. The mean ratio of the PV between the two sites was 7.99 ± 3.04 in the asymptomatic group and 6.22 ± 1.43 in the symptomatic group. The PV ratios (*p* = 0.035) were significantly higher in asymptomatic children with NCS than in the symptomatic subjects (Table 2) (Figure 2). In the symptomatic and asymptomatic groups, neither proteinuria nor BMI were correlated with the ratio of the PV and diameters between the two sites of the LRV.

## 4. Discussion

Renal Nutcracker Syndrome, also recognized as NCP or LRV syndrome, is caused by compression of the LRV between the SMA and the aorta. LRV has many developmental variants; the two most common are the circumaortic and the retrocaval. Anterior NCS is the compression of the LRV between the aorta and SMA, whereas posterior NCS occurs between the vertebral column and the aorta. There are also rare subtypes of NCS, including the compression of the LRV by dilated left-sided inferior vena cava. Other rare variants of NCS include right-sided NCS [2,3,4,19,20]. The nutcracker anatomy is not always associated with clinical symptoms, and some of the anatomic findings suggestive of nutcracker may represent a normal variant referred to NCP. The term NCS is the clinical the same of NCP characterized by a complex of symptoms with substantial variations [4,20,21].

Clinical features of patients with NCS vary from asymptomatic microhematuria to severe pelvic congestion syndrome. Abdominal pain, left flank pain, and macroscopic hematuria is the most commonly symptoms in NCS. Pelvic congestion syndrome characterized by symptoms of lower abdominal pain, dysuria, dysmenorrhea, dyspareunia, pelvic, vulvar, gluteal or thigh varices, and emotional disturbances. This syndrome can also cause lower limb varices and varicoceles. Systemic manifestations have also been reported in adolescents including headache, tachycardia and abdominal pain represented the clinical symptoms of an orthostatic disturbance [2,3,4,6,19,20,21].

We grouped the NCS patients according to the presenting clinical symptoms as symptomatic (loin pain, macroscopic hematuria or both) and non-symptomatic (microscopic hematuria and proteinuria were detected incidentally). Then, we compared their DUS results to determine whether or not there was an association between clinical symptoms and DUS findings among these patients. Our study demonstrated that more than half of the patients with NCS were asymptomatic, and almost half of the NCS diagnoses were detected during proteinuria evaluation. Similarly, near to a half of our patients were symptomatic, and the leading symptom was flank pain.

Nutcracker syndrome is difficult to diagnose and is often a diagnosis of exclusion other possible causes compatible with the clinical presentation of the patient [2,3,5,6,13]. The gold standard methods to identify NCS are still venography of the LRV alongside measurement of the pressure gradient between the LRV and the inferior vena cava, or with intravascular USG [2,13,21]. However, it has been reported that the measurement of the diameter of LRV and the peak flow velocity with DUS may be used with significant success for the diagnosis [2,3,8,11,15,16,22]. According to a study by Kim et al. [15], first applied renal DUS to adults with NCS confirmed by venography and reported that a ratio of AP diameter and PV greater than 5.0 have the sensitivity of 80%, specificity of 94%, and accuracy of 83%. Park et al. [8] proposed that cut-offs >4.2 for the ratio of the AP diameter and 4.0 for the ratio of the PV should be used as sonographic criteria in diagnosing childhood NCS. Takebayashi et al. [16] reported that detection of collateral veins around the LRV on color DUS is a reliable criterion for the diagnosis of NCS. Based on these studies, we think that DUS is the easiest screening method that can be applied for dynamic imaging. In earlier publications [4,21], CTA is accepted as confirmatory imaging techniques in place of conventional venography. It provides a noninvasive evaluation of renal vasculature and retroperitoneal anatomy, as well as the collateral veins. Furthermore, CTA is a faster examination than retrograde venography or MRA. Although CTA, MRA provide a good quality diagnosis of this syndrome, it is very expensive and hard to apply to all patients with suspected NCS as a first-line diagnostic tool. To confirm the diagnosis, we performed DUS first and successfully obtained the DUS images in 36 of 44 patients. Because of the inadequate visualization, 8 patients underwent CTA for the diagnostic purposes along with DUS. 

In our study, the mean ratio of the diameter was 4.36 ± 1.55 and the mean ratio of the PV between the two sides of the LRV was 7.32 ± 2.68, similar to published reports (11,12,15,17,20). When we measured the PV ratios in the clinically symptomatic group, the level was 6.22 ± 1.43 and the diameter ratio between the two portions was 3.69 ± 1.45, which were significantly lower than in the asymptomatic patients. We were expecting a higher levels of PV ratio in the symptomatic NCS patients but clinical asymptomatic group (microscopic hematuria and proteinuria) had a significantly higher PV and diameter ratio. Kim et al. [15] found that the PV at the hilar portion in adults with hematuria with NCS was significantly lower than in normal adults, but Cheon et al. [17] reported no difference between the NCS and normal children. Association of radiologic compression of the LRV with clinical symptoms still remains challenging. In a more recent publication, Hangge et al. [21] studied 33 patients with NCS and 103 controls concluded that the degree of compression of the LRV in NCS was significantly higher compared to controls. The study found a strong correlation between the degree of LRV compression on imaging in diagnosing NCS. The results of our study showed higher compression was associated with microscopic hematuria and proteinuria. Our findings might vary depending on different or complex collateral connections that influence the spread of the LRV pressure in patients. We assume that well-developed renal vein varices and collateral connections effectively drain the renal vein and decrease venous pressure that might result in clinical asymptomatic.

NCS in young adults is generally managed conservatively [2,3,6,11]. In children, the best option is a conservative approach because spontaneous remission at rates of 75% have been reported with growth or weight gain [2,3,11,13,23]. More recently, Alaygut et al. [11] evaluated the clinical findings of 23 children with NCS. The authors concluded that the increase of BMI can influence and regress the symptoms of hematuria and proteinuria. Surgical intervention is reserved for symptomatic and disabling NCS with severe persistent or recurrent hematuria causing anemia and blood clots, which result in abdominal or flank pain [2,5,13]. Options include LRV transposition, LRV bypass, SMA transposition, and renal auto transplantation [2,3,4,5,6,10,13,24,25]. Alternative surgical techniques have become available such as endovascular treatment [3,25], which is a less invasive percutaneous procedure. Endovascular treatment options include embolization, balloon angioplasty, and stenting [25]. As seen in our study, one patient underwent drastic measures for her symptoms without resolution and eventually underwent venous transposition (BMI: 15.8, PV ratio: 4.6); the others did not require any intervention and were managed conservatively. Radiographic data of LRV compression is not essentially to determinate the surgical intervention [4,21]. Higher PV ratios do not necessarily correlate with symptomatology as seen in our patient. 

Our study has some limitations. Our institution is one of the major referral pediatric nephrology center in the north of Turkey and this syndrome is not commonly seen. Therefore, the analysis was examined for a period of seven years and diagnosis was confirmed in a total of 44 pediatric patients with NCS. We did not perform left renal venography, because most of our patients had mild renal symptoms, such as asymptomatic microscopic hematuria detected through mass urinary screening.

## 5. Conclusions

Our study identifies that increased compression ratio of the LRV entrapment is most observed in orthostatic proteinuria and microscopic hematuria. Furthermore, NCS should be considered in the differential diagnosis of young patients with left flank pain. Renal DUS can be recommended as a useful first-choice non-invasive diagnostic technique to confirm LRV compression. 

## Figures and Tables

**Table 1:** Baseline clinical characteristics and left renal vein (LRV) Doppler ultrasonography (DUS) findings of the study cohort.

Variables	Study Population (*n* = 44)
Age (years)	12.8 ± 2.79 (7–18)
Gender, *n* (%)	female, 25 (56.8%), male, 19 (43.2%)
Clinical findings, *n* (%)	
Complaints (negative) *n* (%)	27 (61.4%)
Incidental finding of microscopic hematuria, *n* (%)	6 (13.6%)
Incidental finding of proteinuria, *n* (%)	21 (47.7%)
Complaints (positive), *n* (%)	17 (38.6%)
Loin pain, *n* (%)	9 (20.5%)
Macroscopic hematuria, *n* (%)	6 (13.6%)
Loin pain + macroscopic hematuria, *n* (%)	2 (4.5%)
BMI (kg/m^2^)	16.4 (14.2–26.8)
24-hour urine protein excretion (*n* = 35)	13 (1.6–42)
Individual LRV Doppler US findings	
Diameter at the hilar portion (mm)	8.43 ± 3.25
Diameter at the AM portion (mm)	2 (1.1–6.1)
Diameter Ratio between hilar/AM	4.36 ± 1.55
PV at the AM portion (cm/s)	144.9 ± 56.73
PV at the hilar portion (cm/s)	19.72 ± 5.09
PV ratio between AM/hilar	7.32 ± 2.68
Time to diagnosis (months)	2.50 (1–60)
Treatment, *n*	
Conservative	43
Surgery	1

Data are presented as means ± standard deviations (x ± SD) or as median with range. BMI = body mass index, LRV = left renal vein, PV = peak velocity, AM = aortomesenteric. Complaints (negative) *n* (%): consisted of (Incidental finding of microscopic hematuria, *n* (%), Incidental finding of proteinuria, *n* (%)); Complaints (positive), *n* (%): consisted of (Loin pain, *n* (%) and Macroscopic hematuria, *n* (%)).

**Table 2:** Renal Doppler findings of the left renal vein in the asymptomatic/symptomatic group.

Individual LRV Doppler US Findings	Asymptomatic * Group	Symptomatic ** Group	*p*
Diameter at the hilar portion (mm)	8.93 ± 3.82	7.63 ± 1.89	0.215
Diameter at the AM portion (mm)	2 (1.20–6.10)	2.15 (1.10–4)	0.103
Diameter Ratio between hilar/AM	4.79 ± 1.47	3.69 ± 1.45	0.025
PV at the AM portion (cm/s)	150.1 ± 63.53	135.9 ± 43.01	0.118
PV at the hilar portion (cm/s)	18.77 ± 5.22	21.36 ± 4.55	0.449
PV ratio between AM/hilar	7.99 ± 3.04	6.22 ± 1.43	0.035

Data are presented as means ± standard deviations (x ± SD) or as median with range. * Asymptomatic: Microscopic hematuria, and proteinuria. ** Symptomatic: Left flank pain, macroscopic hematuria, or both.
